# Residential Exposure to Traffic Noise and Health-Related Quality of Life—A Population-Based Study

**DOI:** 10.1371/journal.pone.0120199

**Published:** 2015-03-13

**Authors:** Nina Roswall, Vibeke Høgh, Pernille Envold-Bidstrup, Ole Raaschou-Nielsen, Matthias Ketzel, Kim Overvad, Anja Olsen, Mette Sørensen

**Affiliations:** 1 Danish Cancer Society Research Center, Copenhagen, Denmark; 2 Aalborg AF Study Group, Department of Cardiology, Aalborg University Hospital, Aalborg, Denmark; 3 Department of Environmental Science, Aarhus University, Roskilde, Denmark; 4 Department of Public Health, Section for Epidemiology, Aarhus University, Aarhus, Denmark; The Ohio State University, UNITED STATES

## Abstract

**Background:**

Few studies have investigated the association between objectively measured traffic noise and health-related quality of life. However, as traffic noise has been associated with both cardiovascular disease and diabetes, and health-issues including sleeping problems, annoyance, and stress, it seems plausible that traffic noise is associated with health-related quality of life.

**Methods:**

Between 1999 and 2002, a cohort of 38,964 Danes filled in the short form-36 (SF-36) questionnaire. Residential exposure to road traffic and railway noise was calculated for all historical addresses for 10 years preceding the SF-36, using the Nordic prediction method. Associations between noise exposure and SF-36 summary scales and the eight sub-scales were calculated using general linear models, adjusted for age, sex, socioeconomic status, and lifestyle.

**Results:**

Models adjusted for age, sex and socioeconomic factors showed that a 10 dB higher road traffic noise 1 year preceding SF-36 assessment was associated with a 0.14 lower mental component summary (MCS) score (95% confidence interval (CI) -0.26, -0.01). However, further adjustment for lifestyle factors (smoking, alcohol, and waist circumference) attenuated the association: (-0.08 (95% CI: -0.20, 0.04)). Exposure to more than 55 dB of railway noise in the same time period was borderline significantly associated with lower MCS. The physical component summary was not associated with traffic noise.

**Conclusion:**

The present study suggests a weak association between traffic noise exposure and the mental health component score of SF-36, which may operate through lifestyle. The magnitude of effect was, however, not clinically relevant.

## Background

Exposure to traffic noise has been associated with a number of illnesses, including cardiovascular disease[1,2], and diabetes[3], as well as a range of other health issues, such as stress, annoyance, and sleep disturbance[1,4–7]. According to the WHO, one in three EU citizens is annoyed by environmental noise and one in four report experiencing sleep disturbance due to this[7]. At an individual level, these non-clinical effects have been suggested to hamper an optimal physiological and mental functioning, and thus affect quality of life[1,5].

Quality of life is a broad, multidimensional concept, and epidemiologic studies therefore often focus specifically on health-related quality of life (HRQoL), which is a well-accepted construct defined to capture the effect of factors that affect health—both physically and mentally[8]. It is a subjective measure, which is often assessed by the Medical outcomes study 36-item short form health survey (SF-36) questionnaire, which is a highly validated, generic measure of physical and mental health status[9].

Few studies have investigated effects of different types of traffic noise on quality of life. In general, airport noise has been found more annoying, and with stronger effects on sleep, than road and railway noise[10]. For airport noise, a negative association with quality of life has been suggested in both adults, [11,12] and children[13]. Studies on road traffic and railway noise and quality of life have found mixed results. Two approaches to estimate exposure have been used, namely the subjective perception of noise evaluated by each respondent themselves, and objective measurements of traffic noise exposure, either directly measured or modelled. Studies on subjective exposure, i.e. noise annoyance, tend to find that with a higher level of noise annoyance, people report a poorer quality of life[14–16], whereas studies on objectively measured noise have found mixed results: Some studies report that a higher noise exposure is associated with a poorer both physical and mental health[17–20], whereas one study using the SF-12 found this for women in relation to physical, but not mental health, and they found no association for men[21]. And one study found an association between traffic noise and residential satisfaction, but not overall life satisfaction[22].

The above-mentioned studies are relatively small, with the largest cohort including 6,533 participants[21]. Also, most studies have only estimated noise exposure at one point in time[11,12,14–16,18–22], and are therefore not able to address effects of long-term exposure. The objective of the present study was to fill out these gaps by conducting a large, population-based study of long-term residential road and railway traffic noise and HRQoL.

## Methods and Material

### Study population

The study is based on the prospective Diet, Cancer and Health cohort, which has been described in detail previously[23]. Briefly, 160,725 Danes were invited to participate from 1993–97. Inclusion criteria were 50–64 years of age, residence in the greater Copenhagen or Aarhus area and no previous cancer diagnosis in the Danish Cancer Registry. 57,053 participants accepted the invitation and were included into the study, representing 7% of the Danish population in this age group.

At baseline, participants filled in a food frequency questionnaire and a lifestyle questionnaire, and anthropometric measures were collected by trained personnel. In 1999–2002, participants received a follow-up questionnaire regarding diet, lifestyle and HRQoL, and gave self-reported anthropometric data. In total, 45,271 persons (79% of the original cohort) filled in this second questionnaire, and were available for the present study. Reasons for non-participation were death (14.6%), emigration (3.8%), and no reply to the questionnaire (81.7%).

The study was approved by the local ethical committees of Copenhagen and Frederiksberg Municipalities (in Danish: “*Den Videnskabsetiske komite for Københavns og Frederiksberg Kommuner*”) Approval no.: (KF) 01–345/93. All participants provided written informed consent, and the study was conducted according to the Helsinki Declaration.

### Exposure assessment

Residential address history was collected for all cohort members from 1988 and until follow-up using the Danish civil registration system. Road traffic noise exposure was calculated using SoundPLAN (http://www.soundplan.dk/), which implements the joint Nordic prediction method for road traffic noise[24]. Equivalent noise levels were calculated for each address in a position on the most exposed facade of the building using the following input variables: point for noise estimation (geographical coordinate and height (floor) for each residential address), road links (information on annual average daily traffic, vehicle distribution, travel speed, and road type) and building polygons for all Danish buildings provided by the Danish Geodata Agency. We obtained traffic counts from a national road and traffic database. This is based on a number of different traffic data sources ranked as: 1) Collection of traffic data from the 140 Danish municipalities with most residents, covering 97.5% of the addresses included in the present study. Included roads typically have more than 1,000 vehicles per day and are based on traffic counts as well as estimated/modeled numbers. Traffic data represents the period from 1995–1998; 2) Traffic data from a central database covering all the major state and county roads; 3) Traffic data for 1995–2000 for all major roads in the Greater Copenhagen area; 4) Smoothed traffic data for 1995 for all roads based on a simple method where estimated figures for distribution of traffic by road type and by urban/rural zone are applied to the road network and subsequently calibrated against known traffic data at county level.

No information was available on noise barriers or road surface. Road traffic noise was calculated as the equivalent continuous A-weighted sound pressure level (L_Aeq_) at the most exposed facade of the dwelling at each address for the day (L_d_; 07:00–19:00 h), evening (L_e_; 19:00–22:00 h) and night (L_n_; 22:00–07:00 h), and was expressed as L_den_ (den = day, evening, night). A 5 and 10 dB penalty was applied to evening and night respectively. All values < 42 dB were set to 42 dB, as done in previous studies[25], because we considered this a lower limit of ambient noise.

Railway traffic noise exposure was calculated for all present and historical addresses using SoundPLAN, implementing a Nordic calculation method for predicting noise propagation for railway traffic noise (NORD2000). The input variables for the noise model were: point for noise estimation (geographical coordinate and height), railway links (information on summarized train length, train types, travel speed) and building polygons for all Danish buildings. All noise barriers along the railway are included in the model. Railway traffic noise was expressed as L_den_ at the most exposed facade of the dwelling.

For the assessment of both road and railway traffic noise the terrain was assumed flat, a reasonable assumption in Denmark. Urban areas, roads, and areas with water were assumed to be hard surfaces, whereas all other areas were assumed acoustically porous.

### Outcome

The follow-up questionnaire included the SF-36 questionnaire in Danish. This contains 36 items, addressing eight dimensions of health: physical functioning, role limitations due to physical health problems, bodily pain, general health, vitality, social functioning, role limitations due to emotional problems, and mental health. Each dimension is scored ranging from 0 (worst state of health) to 100 (best state of health). From these dimensions, two summary scales; physical component summary (PCS) and mental component summary (MCS), can be derived using algorithms specified by the developers. These summary scales are standardized to have a mean of 50 and standard deviation of 10, with a score above 50 representing an above-average health and a score below 50 representing a below-average health[9]. Participants missing one or more questions in SF-36 were excluded from the study.

HRQoL is a multi-dimensional concept that includes the physical, psychological, and social functioning associated with an illness or its treatment[26]. It is often assessed using SF-36, which is a validated, generic measure assessing physical and mental health status, but which has also become widely accepted as an indicator of HRQoL because it taps into the individual’s functioning and the quality of life that this may apply.

### Covariates

The selection of covariates was done *a priori* based on existing literature and biological plausibility. The included confounders were measured at time of SF-36. Sex, age, smoking status (never, former, current), gram alcohol/day (continuous), and self-measured waist circumference (continuous) were collected as questionnaire data. Education (basic, vocational, higher), cohabiting status (widowed or longest-living of two partners, divorced or annulled same-sex marriage, married or registered partnership, unmarried), and disposable income (household income after taxation and interest, adjusted for number of persons in the household and divided into tertiles) was collected by linking the personal identification number of each participant to the nationwide register Statistics Denmark, which include yearly information since 1980 from the taxation authorities, the register for Education statistics, the Register for Unemployment, and a Company Register for all companies with more than one employee. Finally; models of road traffic noise were adjusted for railway noise and vice versa.

Furthermore, we used information on Charlson Comorbidity Index in the stratified analyses [27], calculated based on National Patient Registry.

NO_x_ exposure was calculated with the Danish AirGIS dispersion modeling system for the same years as exposure to traffic noise, for all addresses where each individual had lived, as previously described in details[28]. Adjustment for NO_x_ did only result in minor changes in the estimates, and it was therefore not included in the final model.

### Statistical Methods

Linear regression models were used to examine the association between residential traffic noise and PCS, MCS and the eight sub-scales. To test the robustness of our models, we calculated three different versions, with increasing level of adjustment: model 1 with adjustment for age and sex; model 2, as model 1 plus adjustment for socioeconomic factors and railway noise; and model 3 as model 2 plus individual lifestyle factors.

All continuous variables were evaluated by investigating linearity both graphically and by linear spline models. We found significant deviation from linearity for age, alcohol and waist circumference, and these were therefore included in the model as splines with boundaries at 60 years (age), 2 g/day (alcohol), and 78 centimeters (waist circumference).

For train noise, we calculated both linear estimates and categorical analyses with three categories (0 dB, <55 dB and ≥55 dB). This allowed us to examine the estimate for the large proportion of the study population, which had no train noise exposure (80.5%). The use of 55 dB as cut-point was chosen based on consistent use in existing literature.

Effect modification by sex, age, education, Charlson Comorbidity Index (0, 1+) and railway noise exposure was examined by including an interaction parameter in the models, and tested by the Wald test.

P-values < 0.05 were considered statistically significant. Results for railway and road traffic noise are reported as changes in the dependent variable per 10 dB increase in noise exposure, with corresponding 95% confidence intervals (CI). Results for railway noise are also reported as categorical results, to allow comparison between those with no exposure and groups of exposed.

All analyses were performed using SAS 9.3 (SAS Institute Inc., Cary, NC), except for the graphical representation of the association between traffic noise and PCS/MCS score, which was produced using restricted cubic splines in the rms library of the R software (version 2.13.1).

## Results

In total, 45,271 persons, who filled in the follow-up questionnaire, were available for the present study. Of these, 384 persons were excluded, because they had a diagnosis of cancer before baseline, 5,662 were excluded due to missing information on either exposure variables, outcome variables or included covariates, and 261 were excluded because they were exposed to airfield/air-traffic noise. This rendered an analytical cohort of 38,964 persons with complete information on all included covariates.

Table 1 shows the distribution of covariates among all participants, and dichotomized according to the two SF-36 component summary scores. A higher score on the PCS and MCS corresponds to a higher HRQoL. For both scores; those with a score below the median were more likely to be women, smokers, unmarried, and have a shorter education, a lower disposable income, and a higher Charlson Comorbity Index score. Those with a low PCS score were older and had a higher waist circumference, whereas those with a low MCS score were slightly younger and had a lower waist circumference.

**Table 1:** Characteristics of the DCH cohort at time of follow-up by Physical Component Summary (PCS) and Mental Component Summary (MCS) score.

	Entire cohort	PCS≤Median^**a**^	PCS>Median^**a**^	MCS≤Median^**a**^	MCS>Median^**a**^
	N = 38,964	N = 19,500	N = 19,464	N = 19,535	N = 19,429
Women, %	53.3	57.7	48.8	58.0	48.5
Age (years)	61.4 (56.0–69.5)	62.1 (56.1–69.7)	60.7 (56.0–69.2)	61.0 (56.0–69.5)	61.8 (56.1–69.5)
Smoking status, %					
*Never*	38.8	36.2	41.4	37.8	39.8
*Former*	36.4	37.1	35.8	35.1	37.8
*Current*	24.8	26.7	22.8	27.1	22.4
Alcohol, g/day^b^	12.8 (1.2–63.4)	11.7 (0.9–64.4)	13.7 (1.6–62.6)	11.8 (0.7–64.7)	13.3 (1.5–62.7)
Waist circumference (cm)	93.0 (74–114)	94.0 (75.0–117)	92.0 (74.0–110)	92.0 (74.0–114)	94.0 (75.0–113)
Education, %					
*Basic*	24.9	28.8	21.1	26.9	22.9
*Vocational*	45.8	45.5	46.1	44.0	47.6
*Higher*	29.3	25.7	32.8	29.1	29.5
Disposable income					
*First tertile*	16.6	20.0	13.1	19.4	13.7
*Second tertile*	28.7	30.6	26.9	29.3	28.1
*Third tertile*	54.7	49.4	60.0	51.3	58.2
Cohabitation status, %					
*Widowed or longest-living of two partners*	7.6	8.2	7.0	8.3	7.0
*Divorced or annulled same-sex partnership*	14.5	16.1	13.0	16.9	12.2
*Married or registered same-sex couple*	72.2	69.7	74.8	68.3	76.1
*Unmarried*	5.6	6.0	5,2	6.5	4.8
Charlson Comorbidity Index, %					
*0*	82.7	75.7	89.7	80.4	85.0
*1*	10.9	15.0	6.8	12.0	9.8
*2+*	6.4	9.3	3.5	7.6	5.2
L_den_ at time of SF-36 (dB)	56.7 (48.7–70.5)	56.9 (48.8–70.6)	56.3 (48.7–70.3)	57.0 (48.7–70.6)	56.4 (48.8–70.3)
Exposed to train noise, %	19.5	20.0	19.1	19.9	19.2
NO_x_ at time of SF-36 (μg/m^3^)	15.0 (10.1–67.8)	15.0 (10.1–69.2)	14.9 (10.1–65.9)	15.0 (10.1–69.3)	14.9 (10.1–66.0)

^a^ Percentages or Median (5–95% CI)

^b^ Among those drinking alcohol

**Fig 1:**
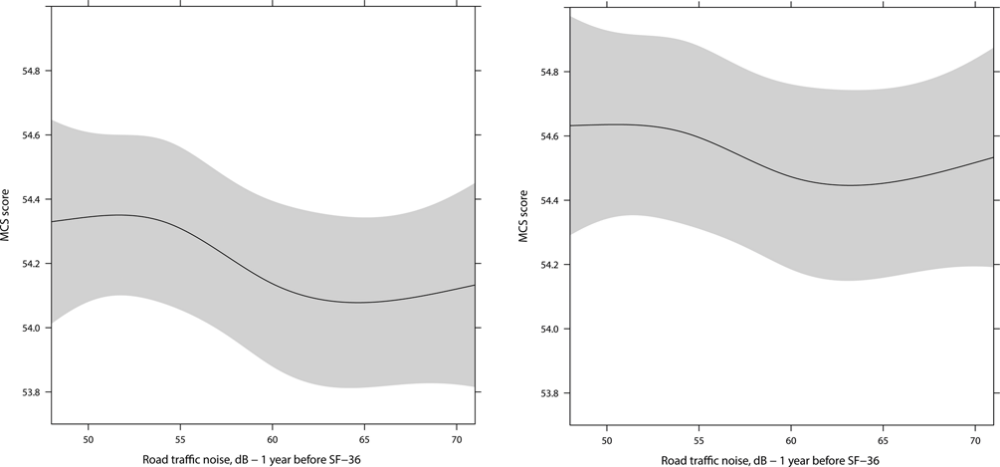
Association between residential exposure to road traffic noise 1 year before SF-36 and MCS score. Left: Model 2, right: Model 3. Development in MCS-score over the spectrum of road traffic noise exposure from 48 to 71 dB. X-aksis: Road traffic noise in dB, y-aksis: MCS-score. Solid line: Estimate. Grey lines: 95% CI.

The Pearson correlation coefficient between road traffic noise 1 and 10 years preceding HRQoL was 0.91. The crude analyses (Model 1) showed statistically significant associations between road traffic noise and both PCS and MCS, suggesting that traffic noise had a negative effect on HRQoL (all p < 0.0001). The models adjusted for socioeconomic factors (Model 2) showed an association between road traffic noise and MCS with a 0.14 lower MCS score (95% CI: -0.26, -0.01) per 10 dB higher road traffic noise 1 year preceding SF-36. The exposure-response curve found no specific window of effect, and showed that the magnitude of effect of traffic noise on HRQoL was not of clinical relevance—especially not after adjustment for individual lifestyle factors (Table 2, Fig. 1). PCS was not significantly associated with SF-36 in the adjusted models (Table 2).

**Table 2:** Crude and adjusted associations between residential road traffic noise exposure (L_den_) and SF-36 component summary scores, per 10 dB.

	*Model 1* ^*a*^Change per 10 dB (95% CI)	*Model 2* ^*b*^Change per 10 dB (95% CI)	*Model 3* ^*c*^Change per 10 dB (95% CI)
**L**_**den**_ **1 year before SF-36-assessment**			
PCS	-0.32 (-0.44, -0.20)	-0.02 (-0.14, 0.10)	0.09 (-0.03, 0.20)
MCS	-0.42 (-0.54, -0.30)	-0.14 (-0.26, -0.01)	-0.08 (-0.20, 0.04)
**L**_**den**_ **10 years before SF-36-assessment**			
PCS	-0.45 (-0.58, -0.33)	-0.09 (-0.22, 0.03)	0.03 (-0.09, 0.16)
MCS	-0.48 (-0.61, -0.35)	-0.15 (-0.28, -0.03)	-0.09 (-0.22, 0.04)

^a^ Adjusted for age, sex

^b^ Adjusted for Model 1 plus education, cohabitance status, income and railway noise

^c^ Adjusted for Model 2 plus smoking status, waist circumference, and alcohol intake

In analyses of road traffic noise and SF-36 sub-scales we found a significant association with two sub-scales in Model 2; both suggesting a negative effect of road traffic noise: *General health* (-0.17 (95% CI: -0.30, -0.03)), and *Mental health* (-0.24 (95% CI: -0.37, -0.11)) per 10 dB higher road traffic noise 1 year preceding SF-36. In contrast, in the fully adjusted model, there was a positive association with *Role—Physical*: 0.13 (95% CI: 0.01, 0.25), whereas the association between road traffic noise and *Mental Health* remained negative: -0.18 (95% CI: -0.31, -0.05) per 10 dB higher road traffic noise. The association with *General Health* was attenuated after adjustment for lifestyle (Table 3).

**Table 3:** Crude and adjusted estimates (95% CI) for association between residential road traffic noise exposure (L_den)_ and SF-36 subscales, per 10 dB.

	*Model 1* ^*a*^ Change per 10 dB (95% CI)	*Model 2* ^*b*^ Change per 10 dB (95% CI)	*Model 3* ^*c*^ Change per 10 dB (95% CI)
**L**_**den**_ **1 year before QoL-assessment**			
Physical Functioning	-0.37 (-0.47, -0.27)	-0.07 (-0.17, 0.04)	0.04 (-0.05, 0.14)
Role—Physical	-0.31 (-0.43, -0.18)	0.04 (-0.09, 0.16)	0.13 (0.01, 0.25)
Bodily Pain	-0.30 (-0.44, -0.15)	-0.02 (-0.16, 0.13)	0.07 (-0.07, 0.21)
General Health	-0.50 (-0.63, -0.36)	-0.17 (-0.30, -0.03)	-0.06 (-0.20, 0.07)
Vitality	-0.47 (-0.61, -0.33)	-0.13 (-0.27, 0.02)	-0.02 (-0.16, 0.12)
Social Functioning	-0.28 (-0.38, -0.18)	-0.03 (-0.14, 0.07)	0.02 (-0.08, 0.12)
Role—Emotional	-0.32 (-0.44, -0.20)	0.03 (-0.09, 0.15)	0.10 (-0.02, 0.23)
Mental Health	-0.52 (-0.65, -0.39)	-0.24 (-0.37, -0.11)	-0.18 (-0.31, -0.05)

^a^ Adjusted for age, sex

^b^ Adjusted for Model 1plus education, cohabitance status, income and railway noise

^c^ Adjusted for Model 2 plus smoking status, waist circumference, and alcohol intake

Railway noise was not associated with PCS. For MCS, there was a borderline significant association in the categorical analyses, for those exposed to ≥ 55 dB: -0.33 (95% CI: -0.67, 0.00), suggesting that railway noise was associated with a poorer HRQoL. However, in the continuous analyses, this was non-significant: -0.08 (95% CI: -0.31, 0.15) per 10 dB (Table 4).

**Table 4:** Association between railway noise exposure at time of SF-36 and PCS/MCS.

	*Model 1* ^*a*^ Estimate (95% CI)	*Model 2* ^b^ Estimate (95% CI)	*Model 3* ^*c*^ Estimate (95% CI)
	**Physical Component Summary (PCS)**		
*Not exposed*	0.00 (ref)	0.00 (ref)	0.00 (ref)
*< 55 dB*	-0.06 (-0.30, 0.18)	0.02 (-0.21, 0.26)	0.08 (-0.15, 0.31)
*≥ 55 dB*	-0.42 (-0.75, -0.09)	-0.17 (-0.49, 0.16)	-0.17 (-0.49, 0.14)
*Linear trend*, *per 10 dB*	-0.26 (-0.49, -0.03)	-0.12 (-0.35, 0.11)	-0.15 (-0.38, 0.07)
	**Mental Component Summary (MCS)**		
*Not exposed*	0.00 (ref)	0.00 (ref)	0.00 (ref)
*< 55 dB*	-0.26 (-0.50, -0.01)	-0.16 (-0.40, 0.09)	-0.12 (-0.36, 0.13)
*≥ 55 dB*	-0.56 (-0.88, -0.21)	-0.33 (-0.67, 0.00)	-0.32 (-0.66, 0.01)
*Linear trend*, *per 10 dB*	-0.18 (-0.41, 0.06)	-0.08 (-0.31, 0.15)	-0.10 (-0.33, 0.13)

^a^Adjusted for age, sex

^b^Adjusted for Model 1plus education, cohabitance status, income and railway noise

^c^Adjusted for Model 2 plus smoking status, waist circumference, and alcohol intake

We investigated effect modification for MSC and PSC by sex, age, education, Charlson Comorbidity Index, and train noise exposure, but found no significant interactions (all p ≥ 0.14, results not shown).

## Discussion

The present study investigated the association between residential noise exposure and HRQoL, measured by the SF-36. There was no association with the physical (PCS) or mental (MCS) component summaries in the fully adjusted models, but a significant, negative association with MCS in models adjusted for socioeconomic factors only. There was a suggestion of a negative association between train noise and MCS in the categorical analyses. There was a significant, inverse association between road traffic noise and the *Mental health* subscale both before and after adjustment for lifestyle covariates.

When interpreting the clinical relevance of SF-36 results, differences of 2–3 points on the PCS scale, 3 points on the MCS and 5–10 points on the sub-scales are considered to be minimally important clinical differences[9]. The effect of traffic noise on HRQoL in the present study is thus modest. However, given the widespread nature of the exposure, the public health impact of the association may still be relevant.

The results of the present study add complexity to the findings of previous studies on objectively measured road and railway traffic noise and HRQoL. However, some of the contradicting results may be explained by the modest size of the previous studies, rendering the statistical power rather low. Also, different methods were used to estimate noise, which do not allow direct comparison of results. Different estimations include vehicle traffic density as a proxy for noise exposure[19,21], standardized day/night measurements of noise[17], comparing quiet and noisy urban and rural areas, proximity to motorways, wind turbines, industrial areas and airports[18], and comparing bedroom orientation towards large roads[20]. In contrast, the present study relied on the Nordic Prediction Model, based on a number of different input variables including information on annual average daily traffic, vehicle distribution, travel speed for all Danish road lines with more than 1,000 vehicles per day, as well as all Danish building polygons[24].

The present study design allowed estimation of associations across different exposure windows in time. When comparing the results of the two time-periods included; 1 and 10 years before SF-36, the association between road traffic noise exposure and HRQoL seemed stronger over the longer exposure period, especially in model 1 (PCS: -0.32 vs. -0.45; MCS: -0.42 vs. -0.48) and 2 (PCS: -0.02 vs. -0.09; MCS: -0.14 vs. -0.15), which may suggest that longer term exposure is important in relation to HRQoL. However, the fact that the difference between estimates is largest in Model 1, may also suggest that the long-time exposure information is more confounded than the short-time exposure information. More studies with long-time exposure information are needed in order to investigate this in detail.

Our finding of a lower score on the subscale *Mental health* with increasing traffic noise exposure adds strength to the similar findings in four smaller, cross-sectional studies, which all found an inverse association between road traffic noise and mental health[15,29–31]. We have identified two longitudinal studies examining changes in quality of life in the same population before and after rerouting roads, leading to a change in traffic noise exposure[32,33]. A UK study found no change in mental health after a 3 dB reduction in traffic noise[32]. In contrast, a Swedish study found a significant decrease in psychological symptoms after a 12 dB reduction in road traffic noise[33]. The difference may be explained by the larger magnitude of change in exposure. It has been suggested that while traffic noise seems able to affect psychological symptoms, it does not seem to induce mental illness per se, but rather accelerate development of already latent mental unbalance and disorders[5].

The non-significant, positive association between traffic noise exposure and PCS in the fully adjusted models of our study is unexpected. When examining the subscales individually, it is the scale *Role physical* which contributes to the overall positive association. This suggests that participants with a high noise exposure have fewer problems with their job or other daily activities because of their physical health[9]. Given the large number of analyses, this may be a chance-finding, and as no strong hypothesis support the finding, further research is required. Interestingly, however, this positive association is only found in the fully adjusted model, and not in the model adjusted for socioeconomic variables only.

In general, the direction of the association between road traffic noise and HRQoL differs markedly between model 2 and model 3 in our study. In model 3 we further adjust for lifestyle factors: smoking, alcohol and waist circumference. Only few studies have previously investigated the association between traffic noise and HRQoL, with no strict strategy of which covariates to include. It is possible, that part of the effect of traffic noise on HRQoL actually operates through pathways where traffic noise affects lifestyle, and through this also HRQoL. E.g., a Dutch study found a positive association between traffic noise and smoking[34], and a Swedish study found a positive association between aircraft noise and waist circumferrence[35]. Thus, lifestyle factors could potentially be mediating variables of the association between traffic noise and HRQoL, rather than confounders. This would entail that we overadjust in the fully adjusted models by removing these pathways through which traffic noise potentially operate[36]. Under such a scenario, the results of Model 2 would represent the true association between residential noise exposure and HRQoL.

In order to investigate this further, we tried adding the individual lifestyle factors (waist circumference, smoking, alcohol) to model 2 one at a time, in order to clarify if a specific variable caused the shift in estimates from model 2 to model 3. There was no clear indication that this difference was attributable to one specific lifestyle factor, although adding waist circumference did show the strongest effect on the estimates. This is in line with the above-mentioned study finding an association between noise and obesity[35], and studies finding an inverse association between obesity and HRQoL[37].

The present study includes objective noise assessment only. It has been proposed, that the subjective assessment of noise moderates the association between noise exposure and health as well as quality of life[19,38]. Noise sensitivity seems to be a relatively stable personality characteristic[22,39], attributable to approximately 25% of the population[39], and it affects noise annoyance[19,39]. Noise annoyance is, however, not only determined by acoustic parameters and personality, but also a combination of housing-related variables, and socio-political factors, that influence the attitude towards noise sources[19,22]. We have found no studies comparing subjective and objective noise assessment and quality of life in the same population. However, a study on subjective and objective noise exposure in relation to sleep quality interestingly found that the effects of objectively modeled traffic noise on objectively measured sleep quality was independent of noise annoyance; with the strongest association found among those who did not report to be annoyed by the noise, whereas noise annoyance was found to mediate the association between objective noise exposure and self-reported sleep quality[40]. This may explain the finding of a more consistent association between subjective noise assessment and quality of life[14–16] compared to objective noise assessment and quality of life[17–22], as quality of life is a highly subjective measure. The use of self-reported data on noise exposure has been questioned, as the response is very sensitive to question wording and personal interest[41]. Also, adaption to traffic noise usually occurs in everyday settings, where people learn to ignore habitual noise. They may thus not report annoyance when questioned, but despite this, the ear is still transmitting signals to the nervous system, that may cause bodily reactions[42], which might affect HRQoL. Thus, subjective noise measures may not capture the full effect of traffic noise on HRQoL. By objectively measuring noise, these pitfalls are avoided, though potentially stronger associations between noise exposure and HRQoL in noise-sensitive groups compared to non-sensitive groups may be overlooked.

The strengths of the present study include the study size, which increases the statistical power. Furthermore, we had detailed address-histories for all participants, allowing collection of virtually complete exposure data for different exposure time windows. The Nordic Prediction Model used to calculate exposure is well-known and regularly used in studies of exposure and health. The outcome, HRQoL, was assessed using the well-validated SF-36 questionnaire; one of the most widely used measures of HRQoL. In the present study, we focused on the two component summary scores. This minimized multiple testing. We did, however, also investigate the eight sub-scales, in order to focus on effects on quality of life, which only register on selected sub-scales. A limitation of the study is that the norm-based scoring of the SF-36 data is based on an American standard population, and may thus differ from the Danish population[9]; this might induce misclassification of the study outcome. However, given the fact that this should be independent of exposure misclassification, and thus non-differential, which most often introduces bias towards the null, rather than confounding. We used a validated noise exposure model, but only had information on outdoor exposure at the most exposed façade. We lacked information on how much time each participant spend at home over the course of the day, and data on indoor insulation, window type and bedroom orientation, which may affect the actual noise exposure. The effect of noise on HRQoL may thus be underestimated in the present study. Originally, approximately 160.000 participants were invited to take part in the Diet, Cancer and Health cohort, of which 57.053 accepted. In the present study, 38,964 of these participated; those who accepted taking part in the follow-up study, and who answered all relevant questions. This means, that the association between traffic noise and HRQoL is examined in a selected population, which may hamper generalizability of the findings if participants display a different association than non-participants.

In conclusion, the present study suggests an association between road traffic and railway noise exposure and the mental component summary of SF-36, which may operate through individual lifestyle factors. Despite being significant, however, the association is small and does not seem clinically relevant. There was no association between traffic noise and the physical component summary. Furthermore, the study contributes to a general discussion on the pathways through which the effects of traffic noise operate on HRQoL and encourages careful consideration on which confounders to include in future studies of the association.
